# *CTLA4* enhances the osteogenic differentiation of allogeneic human mesenchymal stem cells in a model of immune activation

**DOI:** 10.1590/1414-431X20154209

**Published:** 2015-05-19

**Authors:** F. Dai, F. Zhang, D. Sun, Z.H. Zhang, S.W. Dong, J.Z. Xu

**Affiliations:** 1 National and Regional United Engineering Laboratory of Tissue Engineering, Department of Orthopedics, Southwest Hospital, Third Military Medical University, Chongqing, China; 2 Department of Biomedical Materials Science, School of Biomedical Engineering, Third Military Medical University, Chongqing, China

**Keywords:** *CTLA4*, Mesenchymal stem cells, Immunomodulatory, Bone tissue engineering

## Abstract

Allogeneic mesenchymal stem cells (allo-MSCs) have recently garnered increasing interest for their broad clinical therapy applications. Despite this, many studies have shown that allo-MSCs are associated with a high rate of graft rejection unless immunosuppressive therapy is administered to control allo-immune responses. Cytotoxic T-lymphocyte-associated protein 4 (CTLA4) is a co-inhibitory molecule expressed on T cells that mediates the inhibition of T-cell function. Here, we investigated the osteogenic differentiation potency of allo-MSCs in an activated immune system that mimics the *in vivo* allo-MSC grafting microenvironment and explored the immunomodulatory role of the helper T cell receptor*CTLA4* in this process. We found that MSC osteogenic differentiation was inhibited in the presence of the activated immune response and that overexpression of *CTLA4* in allo-MSCs suppressed the immune response and promoted osteogenic differentiation. Our results support the application of *CTLA4*-overexpressing allo-MSCs in bone tissue engineering.

## Introduction

Bone marrow-derived mesenchymal stem cells (MSCs) are a type of pluripotent stem cell that possesses the capacity for self-renewal and differentiation into multiple functional cell types (1). Although evidence indicates that MSCs have therapeutic potential in bone tissue engineering and tissue repair and regeneration (2), differences in patient age and the limited number of available autologous MSCs represent major obstacles to their clinical application. Accordingly, due to their stability and ready availability in large quantities, allogeneic MSCs (allo-MSCs) represent an attractive alternative as a seed cell source for bone tissue engineering (2,3).

The low immunogenicity of MSCs is attributable to their expression of major histocompatibility complex (MHC) class I and adhesion molecules including vascular cell adhesion molecule 1 (VCAM-1), intracellular adhesion molecule 1 (ICAM-1), and lymphocyte function-associated antigen 3 (LFA-3) and their lack of expression of MHC class II molecules (e.g., CD40, CD80, and CD86) (4,5). Indeed, a number of studies have indicated that MSCs are immunosuppressive both *in vitro* and *in vivo* (6), exerting a profound inhibitory effect on the proliferation of T cells, B cells, dendritic cells, and natural killer cells. In addition, an array of soluble factors including transforming growth factor (TGF)-β, prostaglandin E2 (PGE2), indoleamine 2,3-dioxygenase (IDO), and inducible nitric oxide synthase (iNOS) have been suggested to mediate the immunosuppressive effects of MSCs. Consistent with their immunosuppressive properties, allo-MSCs have been shown to prolong skin allograft survival in immunocompetent baboons and to prevent the rejection of melanoma tumor cells in immunocompetent mice (7,8). Despite this, many studies have shown that unless immunosuppressive therapy is administered to control the allo-immune responses, allo-MSCs are associated with a high rate of graft rejection (9–13). For example, in a study of rats with a femoral segmental defect, Tsuchida et al. (9) found that treatment with a short-term immunosuppressant increased the survival time of grafted allo-MSCs and enhanced their ability to repair critical bone defects to a degree comparable to that of autologous MSCs. Moreover, increased rejection of allogeneic donor bone marrow (BM) cells following infusion of donor MSCs in a murine model of allogeneic BM transplantation has been attributed to induction of a memory T-cell response by allo-MSCs (10). In this context, it has been shown that tissue damage gives rise to an inflammatory response involving immune cells and is associated with increased fracture healing time and a higher rate of complications (14). Accordingly, we hypothesized that immune system activation may directly affect allo-MSC differentiation potential.

Cytotoxic T-lymphocyte-associated protein 4 (CTLA4) is an important co-inhibitory molecule expressed on activated T cells that mediates T-cell anergy, apoptosis, and clone deletion and is associated with increased immune tolerance (15). The mechanism of action of CTLA4 involves competitive inhibition of CD28 binding with B7 on antigen-presenting cells (APCs), thereby blocking the B7-CD28 costimulatory pathway and inhibiting T-cell activation (15). A fusion protein comprising the extracellular domain of CTLA4 and a modified CH2-CH3 domain of IgG (16) (CTLA4-Ig) has been shown to inhibit T-cell-dependent antibody responses; prolong transplanted organ survival; and induce long-term donor-specific tolerance in transplants of heart, kidney, bone, and skin (17–19). Moreover, we have shown that human bone marrow-derived MSCs (BMMSCs) overexpressing *CTLA4*-Ig differentiate normally into osteoblasts *in vitro* and form bone tissue in xenotransplantation models in the absence of general immunosuppression (5). Accordingly, in the current study, we set out to evaluate the osteogenic differentiation potency of allo-MSCs in an *in vitro*immune activation microenvironment and to characterize the mechanism underlying*CTLA4* promotion of MSC osteogenic differentiation.

## Material and Methods

### Isolation, culture expansion, and flow cytometry analysis of MSCs

MSCs were isolated and cultured using a previously described method (5). Briefly, about 10 mL iliac crest marrow aspirate was collected from healthy volunteers in a syringe containing 3000 U of heparin. Written informed consent was obtained from all volunteers. The marrow sample was diluted with 10 mL phosphate-buffered saline (PBS) and loaded onto 20 mL Ficoll (TBD Corporation, China) with a density of 1.073 g/mL in a 50-mL conical tube. Cell separation was accomplished by centrifugation at 900*g* for 20 min at 20°C. Nucleated cells were collected from the interface, diluted with 20 mL PBS, and centrifuged at 900*g* for 10 min at 20°C. Cells were resuspended in Dulbecco's modified Eagle's medium (DMEM) containing F12 (Hyclone, USA) and 10% fetal bovine serum (FBS; Gibco, USA). Cells were cultured at a density of 7.5×10^6^ cells per 37.5 cm^2^ flask in F12-DMEM containing 10% FBS at 37°C and 5% CO_2_. When subconfluent, cells were detached using 0.05% trypsin-EDTA (Gibco) and subcultured at a density of 1.8×10^5^ cells per 37.5-cm^2^ flask. After the third passage, cells were harvested for detection of surface antigens by flow cytometry. Cells (5×10^5^) were incubated for 30 min with fluorescein isothiocyanate (FITC)-labeled anti-CD105 monoclonal antibody (Sigma, USA) and phycoerythrin (PE)-labeled anti-CD34 monoclonal antibody (Sigma). FITC-labeled anti-IgG2b monoclonal antibody (Sigma) and PE-labeled anti-IgG1 monoclonal antibody (Sigma) were used as isotype controls. Cells were washed with PBS and resuspended in PBS containing 1% formalin and 0.1% bovine serum albumin (BSA, Sigma). Data were acquired on a FACSCalibur platform (BD Biosciences, USA), and CD105-positive/CD34-negative populations were designated as BMMSCs.

### Infection of MSCs with adenovirus containing the*CTLA4*-Ig gene

Recombinant adenoviruses expressing genes encoding human*CTLA4*-Ig (pAdEasy-*CTLA4*-*EGFP*) and EGFP (pAdEasy-*EGFP*) were gifts from Dr. Jun Wu (Third Military Medical University, China). Viral solutions at a titer of 3×10^6^ colony forming units (CFUs)/mL were used to infect MSCs at 70% confluence. Green fluorescent protein (GFP)-positive cells were detected by fluorescence microscopy, and *CTLA4* expression was characterized by flow cytometry and Western blot (see respective sections).

### Preparation of peripheral blood mononuclear cells and establishment of an immune activation microenvironment

Peripheral blood mononuclear cells (PBMCs) were isolated by Ficoll density gradient centrifugation of heparinized peripheral blood collected from healthy human volunteers. PBMCs were cultured in RPMI 1640 medium (Gibco) supplemented with 100 U/mL penicillin and 100 U/mL streptomycin, 2 mM glutamine, and 10% FBS (Gibco). PBMCs were stimulated with 2.5 μg/mL phytohemagglutinin (PHA, Sigma) for 72 h to establish the immune activation microenvironment.

### Detection of CD80 and CD86 expression in PBMCs

PBMCs (5×10^5^) were stimulated with 2.5 μg/mL PHA for 24 h, after which cells were harvested, washed, and stained for 30 min with FITC-labeled anti-CD80 monoclonal antibody (BD Biosciences) and PE-labeled anti-CD86 monoclonal antibody (BD Biosciences). FITC-labeled anti-IgG2b monoclonal antibody (BD Biosciences) and PE-labeled anti-IgG2a (BD Biosciences) were used as isotype controls, respectively. Cells were washed with PBS and resuspended in PBS containing 1% formalin and 0.1% BSA. Data were acquired on a FACSCalibur platform (BD Biosciences) and analyzed using the FlowJo software (FlowJo, USA).

### Cell proliferation and cytokine production

PBMCs (5×10^5^) were cocultured with 1×10^5^ or 5×10^4^ γ-irradiated (30 Gy) MSCs or*CTLA4*-infected MSCs (MSCs-CTLA4) in a U-bottom 96-well plate for 3 days in the presence or absence of 2.5 μg/mL PHA. Cell proliferation was measured using a Cell Counting Kit-8 assay (Dojindo, Japan) according to the manufacturer's instructions. Levels of interleukin (IL)-2 and interferon (IFN)-γ in supernatants were measured by enzyme-linked immunosorbent assay (ELISA) in accordance with the manufacturer's instructions (R&D Systems, USA).

### Osteogenic differentiation of MSCs *in vitro*

*In vitro* osteogenic differentiation of MSCs was performed as described previously (5). Briefly, PBMCs were stimulated with 2.5 μg/mL PHA to establish the immune-activation microenvironment and cocultured with MSCs or MSCs-CTLA4 at a ratio of 5:1 for 72 h in flat-bottom 96-well plates. The culture medium was then replaced with osteogenic medium [F12-DMEM and 10% FBS plus 100 nM dexamethasone (Sigma), 0.05 mM ascorbic acid-2-phosphate (Sigma), and 10 mM β-glycerophosphate (Sigma)], which was replaced every 3 days for 21 days. Osteogenic differentiation was evaluated at day 9 by alkaline phosphatase (ALP) staining using a Sigma FASTTM BCIP/NBT tablet, and at day 21 using Alizarin red S staining. The image was analyzed using the Image-Pro-Plus 6.0 software (Media Cybernetics, USA).

### Real-time polymerase chain reaction (PCR)

Total RNA was extracted using an RNA Pure Separate Extraction kit (BioTeke, China) according to the manufacturer's instructions. cDNA was synthesized using First Strand cDNA Synthesis Kit according to the manufacturer's instructions (Thermo Fisher Scientific, USA). Real-time PCR was performed on an ABI7500 real-time PCR system (Applied Biosystems, USA) using SYBR Premix EX Taq™ II (Takara, Japan). The primers used were as follows:*RUNX2* (125 bp): 5′-AGATGATGACACTGCCACCTCTG-3′ (F), 5′-GGGATGAAATGCTTGGGAACTGC-3′ (R); *ALP*(162 bp): 5′-ACCATTCCCACGTCTTCACATTTG-3′ (F), 5′-AGACATTCTCTCGTTCACCGCC-3′ (R); *Collagen 1* (147 bp): 5′-CCTGGAAAGAATGGAGATGATG-3′ (F), 5′-ATCCAAACCACTGAAACCTCTG-3′(R);*GAPDH* (159 bp): 5′-ACCCATCACCATCTTCCAGGAG-3′ (F), 5′-GAAGGGGCGGAGATGATGAC-3′(R). Relative expression levels were calculated by normalizing to the expression of the housekeeping gene glyceraldehyde 3-phosphate dehydrogenase (*GAPDH*).

### Western blot

Total protein was extracted with radioimmunoprecipitation assay (RIPA) lysis buffer (KeyGEN BioTECH, China) and separated by 8% sodium dodecyl sulfate-polyacrylamide gel electrophoresis (SDS-PAGE), then transferred onto polyvinylidene fluoride membranes (Millipore, USA). After blocking nonspecific protein binding with 5% BSA, blots were incubated with primary antibodies against CTLA4 (1:1000, R&D Systems, USA), Collagen 1 (1:1000, Abcam, UK), Runx2 (1:1000, Cell Signaling Technology, USA) and GAPDH (1:12000, Sanjian, China). After extensive washing with PBS containing 0.1% Triton X-100, membranes were incubated with horseradish peroxidase (HRP)-conjugated secondary antibody (1:5000, Zhongshan, China) for 30 min at room temperature. The signals were visualized by ChemiDoc XRS (Bio-Rad, USA) using an Enhanced Chemiluminescence kit (Amersham Biosciences, UK) and analyzed using the ImageJ2x software (USA).

### Data analysis

Results are reported as means±SD. The statistical significance of differences between groups was analyzed using two-tailed independent Student's *t*-tests. Analyses were performed using the GraphPad Prism 5.0 statistical software package (GraphPad Software Inc., USA). P<0.05 was considered to be statistically significant.

## Results

### CTLA4 expression in MSCs-CTLA4

**Figure 1:**
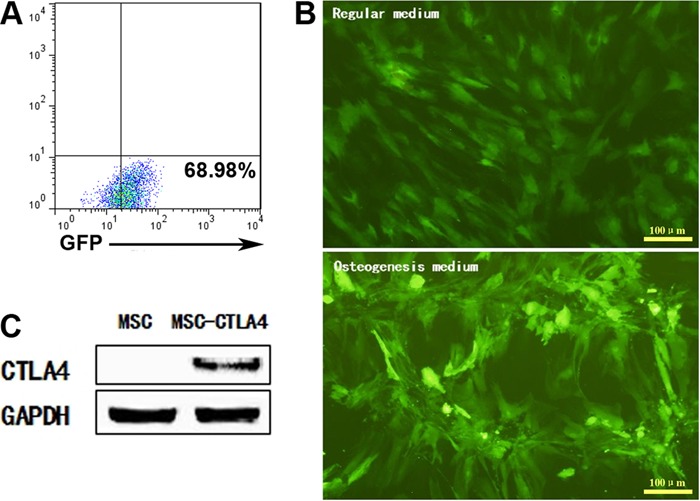
Expression of *CTLA4*-Ig in mesenchymal stem cells (MSCs)-CTLA4. *A*, Flow cytometry analysis of green fluorescent protein (GFP) expression level in MSCs-CTLA4 population shows that the percentage of GFP-positive cells was 68.98%. *B*, Strong fluorescent signal in MSCs-CTLA4 as determined by fluorescence microscopy (magnification 100×).*C*, Western blot shows elevated expression of CTLA4-Ig protein in MSCs-CTLA4.

Fluorescent-activated cell sorting (FACS) analysis demonstrated expression of CD105 and a lack of expression of CD34 in third-passage MSCs, indicating their successful expansion (data not shown). Expression of*CTLA4*-Ig in MSCs was determined by flow cytometry (Figure 1A) and fluorescence microscopy (Figure 1B), which showed that approximately 69% of MSCs-CTLA4 were GFP positive, and 95% of MSCs-CTLA4 exhibited strong fluorescence in both regular and osteogenic media. Western blotting confirmed elevated levels of CTLA4-Ig protein in MSCs-CTLA4 (Figure 1C).

### Effect of MSCs-CTLA4 on T lymphocyte proliferation and activation

**Figure 2:**
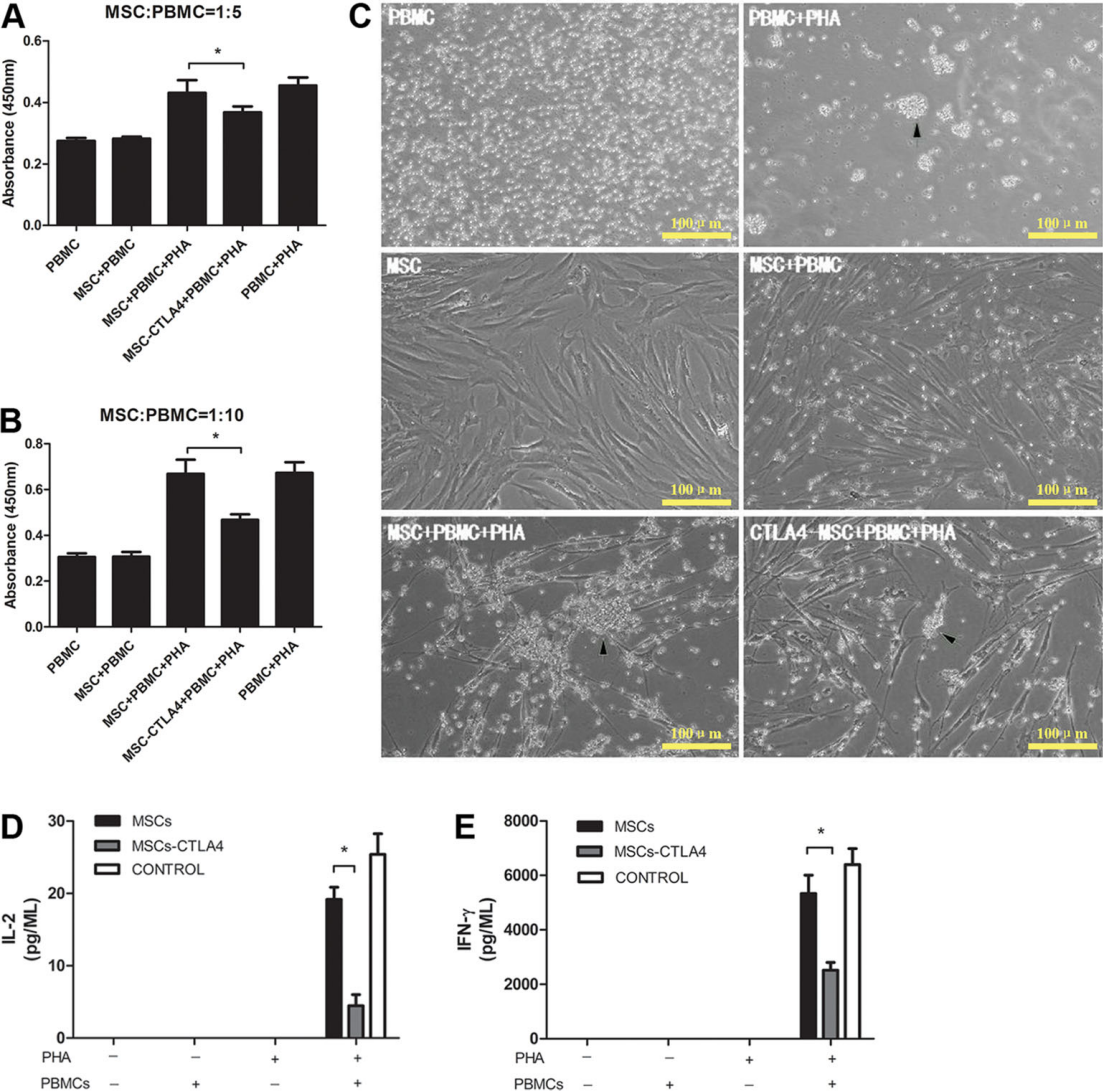
Proliferation and activation of T lymphocytes in the presence of mesenchymal stem cells (MSCs) and MSCs-CTLA4. γ-irradiated MSCs-CTLA4 or MSCs were cocultured with peripheral blood mononuclear cells (PBMCs) at a ratio of 1:5 (*A*) or 1:10 (*B*) in the presence or absence of 2.5 μg/mL phytohemagglutinin (PHA). Cell proliferation was measured by CCK-8 assay. Data are reported as the absorbance values of each group.*C*, Representative light microscope image (magnification 100×). *D* and *E*, Levels of interleukin (IL)-2 and interferon (IFN)-γ in supernatants. Data are reported as means±SD. *P<0.05 (Student's *t*-test).

Next, to investigate the effect of allo-MSCs on T lymphocyte proliferation, γ-irradiated MSCs-CTLA4 or MSCs were cocultured with PBMCs at a ratio of 1:5 or 1:10 in the presence or absence of 2.5 μg/mL PHA. We found that lymphocyte proliferation was inhibited by allo-MSCs-CTLA4 at both ratios in the presence or absence of PHA, but not by allo-MSCs (Figure 2A-C), confirming the inhibitory effect of CTLA4 on T lymphocyte proliferation*in vitro*.

Consistent with this, levels of IL-2 and IFN-γ in the supernatant of MSCs-CTLA4 were significantly lower than in the supernatants of MSCs and PHA-positive control cells (Figure 2Dand E).

### Osteogenic differentiation potency of MSCs-CTLA4 in an immune activation state *in vitro*

**Figure 3:**
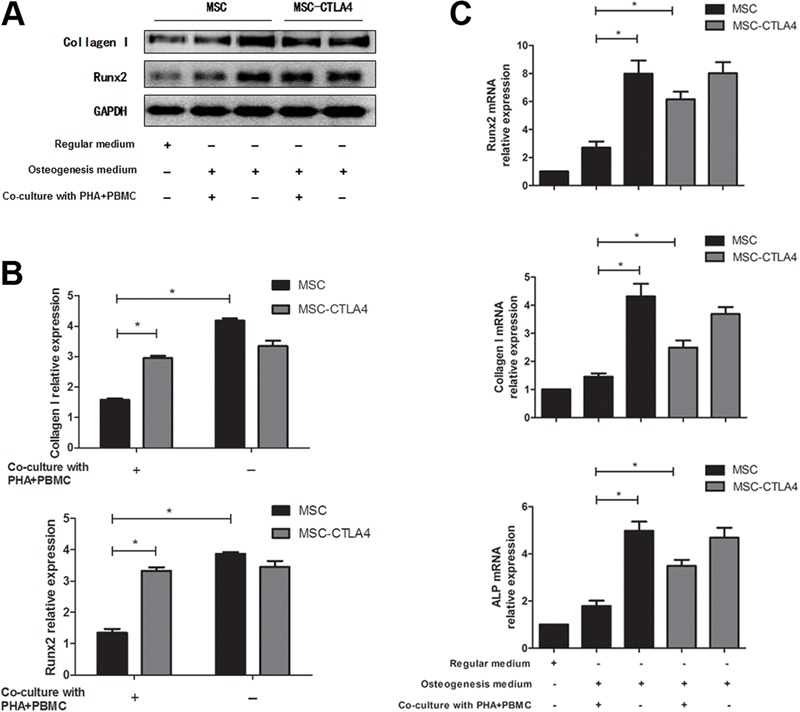
Protein levels and mRNA expression of Runx2, collagen 1 and alkaline phosphatase (ALP). Activated peripheral blood mononuclear cells (PBMCs) were co-cultured with mesenchymal stem cells (MSC) or MSC-CTLA4 at a ratio of 5:1 for 2 h in flat-bottom 96-well plates, after which culture medium was replaced with osteogenic medium and incubated for 21 days.*A* and*B*, Protein levels of Runx2 and collagen 1 after 9 days of culture in osteogenic medium. *C*, mRNA expression levels of Runx2, collagen 1 and ALP after 9 days of osteo-induced cultivation. Data are reported as means±SD. *P<0.05 (Student's *t*-test).

**Figure 4:**
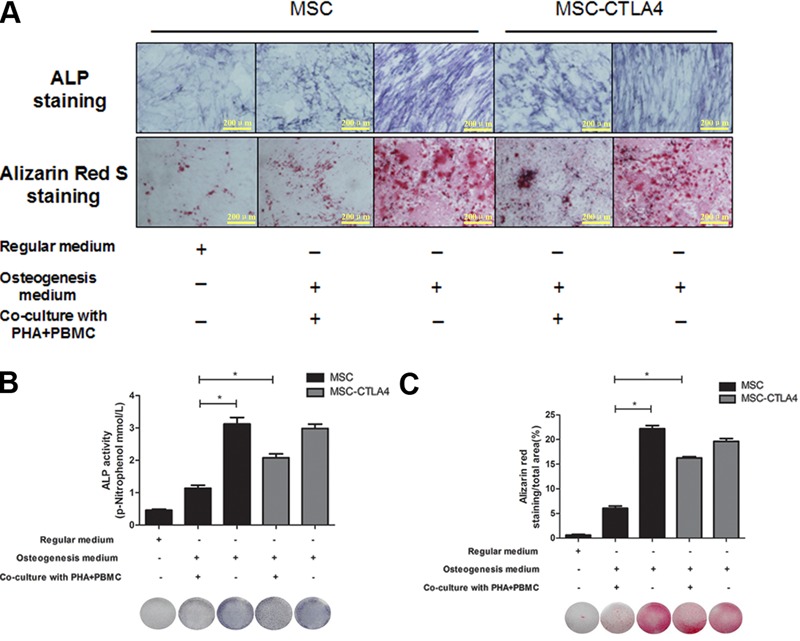
Alkaline phosphatase (ALP) activity and number of calcium nodules after 21-days culture in osteogenic medium. *A*, Images of ALP and Alizarin red S staining. *B*, ALP activity. *C*, Number of mineralized nodules determined quantitatively by Alizarin red S staining. MSC: mesenchymal stem cells; PHA: phytohemagglutinin; PBMC: peripheral blood mononuclear cells. Data are reported as means±SD. *P<0.05 (Student's*t*-test).

Next, we evaluated the effect of *CTLA4* expression on MSC osteogenic differentiation in an immune activation state. MSCs-CTLA4 and MSCs exhibited comparable rates of osteogenic differentiation when cultured in osteogenic medium alone. However, when cells were cocultured with PHA-stimulated PBMCs in osteogenic medium, expression of Runx2, Collagen 1, and ALP were significantly lower at both the protein (Figure 3A and B) and mRNA (Figure 3C) levels in MSCs than in MSCs-CTLA4. Consistent with this, ALP and Alizarin red S staining indicated that both ALP activity and the number of mineralized nodules were significantly lower in MSCs than in MSCs-CTLA4 (Figure 4A-C) cultured under the same conditions. Collectively, these results indicate that MSC osteogenic differentiation is impaired in an activated immune microenvironment and that this inhibition is relieved by *CTLA4*expression.

## Discussion

The current study set out to evaluate functional interactions between allo-MSCs and activated immune cells to better understand the mechanisms of immune-mediated rejection of MSC grafts. To accurately mimic host immune system activation during allo-MSCs grafting, we used PHA, a T-cell activation mitogen, to stimulate the proliferation of PBMC-derived T lymphocytes*in vitro*. We demonstrated, for the first time, that osteogenic differentiation of allo-MSCs was inhibited in this immune-activated state and that overexpression of the helper T cell receptor *CTLA4* in these cells restored their osteogenic differentiation potential in this environment.

Previous studies have demonstrated inhibition of T-cell proliferation by MSCs in mixed-lymphocyte culture or during incubation with mitogens (20–22), indicating that in addition to low immunogenicity, allo-MSCs also possess immunosuppressive properties. Under certain circumstances however, immunosuppression may have negative or undesirable effects such as inhibition of graft implantation. For example, Poncelet et al. (11) reported that, although MSCs had no immunogenicity *in vitro*, they could activate the host immune system *in vivo*. Moreover, infusion of donor MSCs has been shown to induce a memory T-cell response that significantly increased rejection of allogeneic donor BM cells (10). Furthermore, promotion by allo-MSCs implants of host-derived lymphoid CD8^+^T, natural killer T (NKT), and NK cells has been shown to result in immune rejection (12), suggesting that MSCs are not intrinsically immunoprivileged and are unsuitable as universal donors in immunocompetent MHC-mismatched recipients.

We found that levels of the osteogenic markers Runx2, Collagen 1, and ALP, ALP activity, and the number of mineralized nodules were all significantly reduced in allo-MSCs cocultured with PHA-stimulated PBMCs in osteogenic medium compared to allo-MSCs cultured alone (Figures 3 and 4). These results are consistent with previous reports suggesting that allo-MSC osteogenic differentiation is compromised in an activated immune microenvironment. Our data further strengthened the hypothesis that activation of the host immune system during implantation of allo-MSCs might result in limited new bone formation.

Processes invoked to explain immune system activation during grafting include tissue damage-induced inflammation and recognition of implanted scaffold materials as antigens by APCs (23–25). In addition, MSC differentiation into a variety of cell types after implantation has been shown to be accompanied by increased expression of MHC-class II molecules, enhancing their antigenicity and immunogenicity (26). Based on these observations, we hypothesized that activation of the immune system might directly affect the allo-MSC differentiation potential and that immune rejection destroys these seed cells prior to their differentiation into osteoblasts. Accordingly, to promote the acceptance of grafted allo-MSCs, we converted these cells to tolerance-inducing cells by overexpressing the helper T cell receptor*CTLA4*. The CTLA4-Ig fusion protein was previously shown to block the CD80/CD86-CD28 costimulatory pathway to induce antigen-specific immune suppression and to promote osteogenic differentiation of MSCs in xenotransplantation models in the absence of general immunosuppression. Consistent with these reports, *CTLA4* overexpression in allo-MSCs enhanced their inhibition of lymphocyte proliferation and IL-2 and IFN-γ production (Figure 2) and increased lymphocyte expression of CD80 and CD86 (Supplementary Figure S1). CTLA4 also induced the expression of IDO, which acts *in trans* to suppress activation of conventional T cells (27), confirming that*CTLA4* exerts its inhibitory effect on T lymphocytes function*in vitro* by competitively binding with CD28 and blocking the B7-CD28 costimulatory pathway. The other possible molecular mechanism is that*CTLA4* inhibits T cell receptor (TCR) signal transduction by binding to TCRζ and inhibiting tyrosine phosphorylation after T cell activation (28). *CTLA4* has been shown to suppress T cell activation and function via recruitment of the phosphatases SH2 domain-containing tyrosine phosphatase 1 (SHP1), SHP2, and serine/threonine protein phosphatase 2A (PP2A). These phosphatases dephosphorylate several of the major signaling nodes such as nuclear factor of activated T cells (NF-AT), NF-κB, mammalian target of rapamycin (mTOR) and activator protein 1 (AP-1), which are essential for costimulation of T cells (29). Furthermore, *CTLA4* overexpression enhanced the osteogenic differentiation capacity of allo-MSCs in osteogenic medium in the activated immune state, but not under normal osteogenic conditions (Figures 3 and 4).

Our study has a number of limitations. The intricate nature of bone reconstruction involves the interactions of variety of cells *in vivo*, which complicates the development of a suitable bone remodeling and immune microenvironment *ex vivo* (30,31). Indeed, although grafted allo-MSCs have been shown to be capable of prolonged survival, they fail to successfully differentiate into bone tissue *in vivo* (9). Consequently, although*CTLA4* promoted the osteogenic differentiation of MSCs*in vitro*, whether it has similar effects *in vivo* will require further study. Secondly, although adenoviruses achieve more efficient gene transfer than retroviruses and do not integrate the target gene into the host cell genome, their potential tumorigenicity restricts their clinical applications (32). Consequently, additional studies will be required to verify the safety of*CTLA4* overexpression in allo-MSCs *in vivo*.

In conclusion, our results demonstrated that the osteogenic differentiation of allo-MSCs is compromised in an activated immune environment and that expression in these cells of *CTLA4* reversed this inhibition. Our study holds promise for the future success of allo-MSC engraftment in bone engineering and holds out the possibility that *CTLA4*-expressing MSCs can give rise to bone tissue *in vivo*, even in the setting of immune-mediated rejection.

### Supplementary Material

Click here to view [pdf]
